# Perceived Organizational Politics, Engagement, and Stress: The Mediating Influence of Meaningful Work

**DOI:** 10.3389/fpsyg.2019.01612

**Published:** 2019-07-10

**Authors:** Erin M. Landells, Simon L. Albrecht

**Affiliations:** School of Psychology, Deakin University, Melbourne, VIC, Australia

**Keywords:** organizational politics, work engagement, stress, meaningful work, measures

## Abstract

The research aimed to assess proposed associations between organizational politics and employee engagement, employee stress (or more correctly ‘strain’), and work meaningfulness. Very few studies have examined these associations. Confirmatory factor analyses established the dimensionality and reliability of the full measurement model across two independent samples (*N* = 303, *N* = 373). Structural equation modeling supported the proposed direct associations between organizational politics, operationalized as a higher order construct, and employee stress and employee engagement. These relationships were shown to be partially mediated by meaningful work. As such, politics had significant indirect effects on engagement and stress through meaningful work. The results also showed a significant and direct association between stress and engagement. Overall, the results shed important new light on the factors that influence engagement, and identify work meaningfulness as an important psychological mechanism that can help explain the adverse impact of organizational politics on employee engagement and stress. The results also support the dimensionality and validity of a new set of measures of perceived organizational politics focused on generalized perceptions about the use and abuse of relationships, resources, reputation, decisions, and communication channels. More generally, the results serve as a platform for further research regarding the negative influence of organizational politics on a range of individual and organizational outcomes.

## Introduction

**Figure 1:**
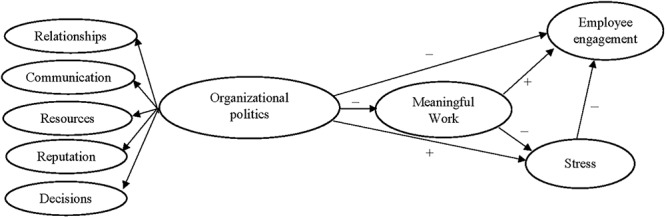
Proposed model.

The detrimental, damaging, and negative effects of organizational politics on outcomes such as stress, burnout, turnover intentions, job satisfaction, and organizational commitment have been well-established with theory and research (Hochwarter et al., 2003; Miller et al., 2008; Chang et al., 2009; Vigoda-Gadot and Talmud, 2010). However, only a limited amount of research has examined the effect of organizational politics on employee engagement, a construct increasingly recognized as important to organizational success and competitive advantage (Macey and Schneider, 2008; Albrecht et al., 2015; Barrick et al., 2015). The present research extends previous research by examining the associations between organizational politics and employee engagement, in addition to associations between organizational politics and stress. The current study also extends past research by assessing these relationships with a newly developed, theory-based, five-dimensional measure of perceived organizational politics. Additionally, and consistent with engagement theory (Kahn, 1990), Job Characteristics theory (Hackman and Oldham, 1976), Job Demands-Resources theory (Bakker and Demerouti, 2014) and relatively recent empirical studies (e.g., Ferris and Treadway, 2012; Rosen et al., 2014; Landells and Albrecht, 2017), the research also examines the potentially important mediating role of work meaningfulness on the proposed associations (Ferris and Treadway, 2012; Rosen et al., 2014; Landells and Albrecht, 2017). The largely untested association between employee stress or ‘strain’ and engagement is also examined (see Figure 1).

### Definitions and Measures of Organizational Politics

Organizational politics has traditionally been defined as behavior that is “self-serving, contradictory to organizational objectives, and premeditated to cause individuals, groups or entities harm” (Hochwarter and Thompson, 2010, p. 1372). Kacmar and Baron (1999) similarly argued that organizational politics “involves actions by individuals that are directed toward the goal of furthering their own self-interests without regard for the well-being of others or their organization” (p. 4). Landells and Albrecht (2016), based on their qualitative research (Landells and Albrecht, 2015), proposed five dimensions of organizational politics that could encompass both positive and negative perspectives: (1) building and using relationships, (2) building personal reputation, (3) controlling decisions and resources, (4) influencing decision-making, and (5) the use of communication channels. These dimensions both overlap with, and extend, previously validated measures of organizational politics (e.g., Kacmar and Ferris, 1991; Drory, 1993; Kacmar and Carlson, 1997; Fedor and Maslyn, 2002; Hochwarter et al., 2003; Vigoda-Gadot et al., 2003). Items in the ‘relationships’ and ‘reputation’ dimensions, for example, have items similar to items in the ‘general political behavior’ and ‘go along to get ahead’ dimensions of Kacmar and Ferris’s (1991) Perceptions of Organizational Politics Scale (POPS) and Hochwarter et al.’s (2003) unidimensional measure. The inclusion of dimensions focused on ‘controlling decisions and resources,’ ‘influencing decision-making,’ and ‘the use of communication channels’ extends the scope of existing measures in that neither the POPS nor the Hochwarter’ and colleagues measures explicitly focus on influencing decisions or gossip. Consistent with most existing measures of organizational politics, the Landells and Albrecht (2016) measure is focused on employee perceptions of the organizational political climate as opposed to personal experiences of politics.

### Outcomes Associated With Organizational Politics

#### Individual Stress

As previously noted, the relationship between perceptions of organizational politics and stress or strain has been confirmed through extensive research (Ferris et al., 1996; Cropanzano et al., 1997; Vigoda, 2002; Treadway et al., 2005b; Miller et al., 2008; Chang et al., 2009; Bedi and Schat, 2013). Miller et al. (2008) meta-analysis, drawing from 24 independent samples and approximately 9000 participants, showed a mean corrected correlation of *p* = 0.45 between organizational politics and stress. Numerous theoretical frameworks such as transactional theory (Lazarus, 1991), conservation of resources theory (Hobfoll, 1989), effort-reward imbalance theory (Siegrist, 2001), appraisal theory (Troup and Dewe, 2002), person-environment fit theory (Caplan, 1987), and demands-control theory (Karasek, 1979) have been proposed to explain the relationship. Consistent with the current focus on the relationship between politics and engagement, Job Demands-Resources (JD-R) theory (Bakker and Demerouti, 2014) has also been invoked to explain that negative organizational politics can operate as a stressor or a ‘hindrance demand’ (Cavanaugh et al., 2000) that can lead to stress and burnout (Albrecht and Landells, 2012). As shown in Figure 1, the current research extends the JD-R framework to additionally examine whether organizational politics is negatively associated with engagement, and whether work meaningfulness partially mediates the relationship between organizational politics and engagement, and organizational politics and stress. It is noteworthy that researchers have cautioned against confusing the word stress with ‘stressor’ or ‘strain’ (e.g., Sonnentag and Frese, 2003). As such, although the authors of the ‘stress’ scale used in the study referred to their construct as ‘stress,’ the measure more accurately reflects what researchers generally refer to as strain. Nevertheless, for present purposes, the terms stress and strain are occasionally used interchangeably.

#### Employee Engagement

Employee engagement has emerged as a construct of interest because it has been shown to be a strong predictor of a range of attitudinal, behavioral, and organizational outcomes (Albrecht, 2010; Christian et al., 2011; Carter et al., 2016). Engagement is often defined as a “positive, fulfilling, work-related state of mind that is characterized by vigor, dedication and absorption” (Schaufeli et al., 2002, p. 74).

As previously noted, although it has been argued that organizational politics can lead to decreased engagement (Byrne et al., 2017; Landells and Albrecht, 2017), only a few researchers have empirically examined the relationship (e.g., Karatepe, 2013; Kane-Frieder et al., 2014; Eldor, 2016). Job Demands-Resources theory (JD-R; Bakker and Demerouti, 2007, 2014) provides a potentially useful explanatory framework within which to examine the association between organizational politics and engagement. In brief, JD-R theory suggests that work engagement, as a motivational construct, mediates the relationships between job and personal resources (e.g., job autonomy, self-efficacy) and positive individual and organizational outcomes (e.g., individual well-being, job performance, competitive advantage). Job demands in the JD-R framework are proposed, via an energy depleting and health impairment pathway, to adversely impact engagement and to be associated with negative individual and organizational outcomes (e.g., depression, absenteeism) through burnout (e.g., Schaufeli, 2013). Consistent with JD-R theory, Crawford et al. (2010) meta-analysis of four samples consisting of 3,042 participants provided evidence of a significant, yet relatively modest, negative association between organizational politics (as a demand) and engagement (*p* = -0.25). By way of explaining the association, it is here proposed that where employees share perceptions that people are undermining and manipulating others, gossiping, and abusing authority, employees will be less energized by and involved with their work. Consequently, Figure 1 shows organizational politics having a direct negative association with employee engagement. Figure 1 also shows a direct negative association between stress and engagement. Although the association between stressors and engagement has been clearly established, somewhat surprisingly, there has been limited research linking the individual experience of stress itself, and engagement. Consistent with arguments that when employees experience stress their energy levels are depleted and engagement is therefore diminished (Hakanen et al., 2006; Schaufeli et al., 2009; Byrne et al., 2017), it is here proposed that stress will be negatively associated with engagement.

#### Work Meaningfulness

Work meaningfulness refers to employees feeling that the work they do is worthwhile, useful, and valuable (Kahn, 1990). Similarly, Albrecht (2015b) defined work meaningfulness as “a positive work-related psychological state reflecting the extent to which employees think and feel they make a significant, important, and useful contribution to a worthwhile purpose in the execution of their work” (p. 212). Researchers have long argued that individuals determine the meaning and value of their work based on cues from their work environment (e.g., Hackman and Oldham, 1976; Kahn, 1990; Ferris et al., 2002; Latham and Pindar, 2005; Rosso et al., 2010; Albrecht, 2013). Humphrey et al. (2007) meta-analysis of job characteristics research identified work meaningfulness as the “most critical” (p. 1341) psychological state and as having a primary influence on work outcomes such as job satisfaction and subjective ratings of performance. In addition to the outcomes included in Humphrey et al. (2007) meta-analysis, meaningfulness has also been theorized and shown to be associated with engagement (Kahn, 1990; May et al., 2004; Albrecht and Su, 2012; Albrecht, 2013; Kahn and Heaphy, 2014; Byrne et al., 2017). May et al. (2004) showed that meaningfulness had a strong positive association with engagement.

With respect to the proposed association between organizational politics and work meaningfulness (see Figure 1), engagement theory (Kahn, 1990) would suggest that perceptions of negative organizational politics (including manipulation, criticism, undermining, disrespect, and disadvantage) can severely impact employees’ willingness to invest themselves in their role and their organization. If employees perceive that their work environment is characterized by gossip, backstabbing, misuse of power, and improper use of relationships, employees may feel the value of their work is unimportant or diminished. In support of this proposed association, Kiewitz et al. (2002) reported a significant association between the POPS and meaningful contribution (*r* = -0.44; *p* < 0.05).

Kiewitz et al. (2002) also examined the harmful effects of negative organizational politics on organizational commitment but did not however, examine the potential mediating effects that derive from job characteristics theory and engagement theory (Bakker and Demerouti, 2014).

Beyond assessing the direct associations between organizational politics and stress, and between organizational politics and engagement, it is important on theoretical grounds to identify the psychological mediating variables that might explain the associations. A limited number of researchers have investigated whether constructs such as psychological needs satisfaction, stress, psychological safety, and morale mediate the relationships between organizational politics and outcomes such as creativity and proactive behavior (Chang et al., 2009; Rosen and Levy, 2013; Li et al., 2014; Rosen et al., 2014). Recently, researchers (e.g., Byrne et al., 2017; Landells and Albrecht, 2017) have theorized that the psychological conditions of psychological availability, safety, and meaningfulness (Kahn, 1990) provide insight into ‘the black box’ explanatory mechanisms that link perceptions of organizational politics and engagement. As previously noted, although researchers have found that the psychological conditions mediate the relationships between job resources and engagement, the propositions that work meaningfulness mediates the associations between organizational politics and both employee engagement and stress remain largely untested. It is here argued that because manipulation, criticism, undermining, disrespect, and disadvantage can severely impact employees’ willingness to invest themselves in their role and their organization (Kahn, 1990), work meaningfulness is likely to be particularly relevant as a mediator of the relationship between politics and engagement. As such, when employees experience manipulative, unfair, and self-serving behavior, they will be less likely to perceive that their work and the work of others makes a valuable contribution and serves a worthwhile purpose, and will therefore likely to be less engaged.

In summary, the study aimed to make a number of contributions to the literature. First, the research aimed to test relationships between newly developed measures of organizational politics and two important aspects or outcomes of the employee experience—employee stress and employee engagement. Furthermore, the research aimed to assess if work meaningfulness acts as a mediating mechanism to, in part, explain the relationships between organizational politics and the proposed outcomes. As shown in Figure 1, it is proposed that work meaningfulness partially mediates the relationships between organizational politics, and both stress and employee engagement. Additionally, the research makes a novel contribution to the literature by assessing the relationship between stress and engagement.

## Materials and Methods

### Item Development and Data Analytic Strategy

To identify items for the proposed measures of perceived organizational politics, an initial pool of items was generated based on the findings of qualitative research (Landells and Albrecht, 2015) and an extensive literature review of published measures and models (Kacmar and Ferris, 1991; Ferris and Kacmar, 1992; Chao et al., 1994; Parker et al., 1995; Kacmar and Carlson, 1997; Buchanan and Badham, 1999; Hochwarter et al., 2003; Ferris et al., 2005; Treadway et al., 2005c; Buchanan, 2008; Fedor et al., 2008; Landells and Albrecht, 2015). The items were designed to assess negative organizational politics across five dimensions. Each of the researchers independently reviewed the potential items, and then agreed on the 18 items that best captured each of the five dimensions: relationships (4 items); reputation (4 items); decisions (3 items); resources (3 items); communication (4 items). All items had the organization as a referent.

In line with Anderson and Gerbing’s (1988) two-step procedure, confirmatory factor analysis (CFA) was first conducted on Sample 1 data to assess the fit of the proposed measurement model and to determine the need for any theoretically defensible respecification. The measurement model was then tested and cross-validated in the second sample to establish the generalizability of the measures. At this stage, tests were also conducted to evaluate the proposed higher order modeling of organizational politics as shown in Figure 1. Structural equation modeling (SEM) of Sample 2 data was then conducted to test the proposed relationships (see Figure 1). The final structural model was then cross-validated using Sample 1 data to help assess the generalizability of the model.

### Participants and Procedure

Respondents in Sample 1 and Sample 2 completed a voluntary on-line survey using procedures approved by both authors’ university ethics committee. The approval was granted in accord with the Australian Government National Statement on Ethical Conduct in Human Research (2007). Before being able to proceed to the survey, all participants clicked a response button confirming they understood the information provided in a participant sheet and confirming they consented to participate in the research. The participant information sheet made clear the anonymity and confidentiality of all responses. No inducements were provided.

Sample 1 data (*N* = 303) were collected through a snowball sampling strategy, drawing on the first author’s professional networks. Participants needed to be at least 18 years old and to have worked in an organization with at least 15 employees for a minimum of 3 months. Participants ranged in age from 23 to 66 years (*M* = 42 years, *SD* = 9 years), were 24% male, 76% female, and had job tenure between 1 and 38 years (*M* = 8 years, *SD* = 7 years). Participants worked in organizations ranging in size from 15 to 250 employees (34% of respondents), 251 to 1000 employees (33%), to more than 1000 employees (33%).

Sample 2 participants (*N* = 353) were employees of a large Australian government organization (2350 staff; 15% response rate). Participants ranged in age from 18 to 80 years (*M* = 41 years, *SD* = 11 years), and included 38.5% males and 60% females (four participants did not indicate their gender). Job tenure ranged from less than a year to 35 years (*M* = 7 years, *SD* = 6 years). Soper’s (2016) SEM on-line calculator demonstrated that both samples exceeded the minimum sample size of 166 to establish sufficient power to test the proposed model.

### Measures

*Organizational politics* was measured, as described above, with 18 items that were developed to measure five proposed dimensions. All items were anchored on a seven-point Likert-type scale (1 = strongly disagree, 7 = strongly agree). *Employee engagement* was measured with the six vigor and dedication items of the 9-item Utrecht Work Engagement Scale (UWES-9; Schaufeli et al., 2006). Acceptable alpha reliabilities have previously been reported for the 6-item engagement scale (e.g., de Lange et al., 2016); a 4-item scale (e.g., Albrecht and Marty, 2017; α = 0.91), and a 3-item scale (Schaufeli et al., 2017; α = 0.77 to 0.95). *Individual stress* was measured with a four-item scale used by Vigoda-Gadot and Talmud (2010; α = 0.75) and adapted from House and Rizzo (1972). *Work meaningfulness* was measured with a scale developed by May et al. (2004) and adapted from Spreitzer (1995). May et al. (2004) reported an alpha reliability of α = 0.90 for the six-item scale.

## Results

### Measurement Models

Using Sample 1 data, CFA was first conducted on the proposed measurement model, with each of the 34 items specified to load on their designated construct. The results yielded only reasonably good fit to the data (see Table 1). Although all standardized loadings were significant, ranging from 0.665 to 0.944, the CFI and the RMSEA point estimate indicated less than acceptable fit.

**Table 1:** Fit indices for alternative measurement and structural models.

Model	χ^2^	*df*	χ^2^/*df*	TLI	CFI	SRMR	RMSEA	RMSEA 90% CI	AIC
Measurement Model Sample 1									
Proposed	1281.937	498	2.574	0.918	0.927	0.059	0.072	0.067–0.077	1457.937
Re-specified	444.544	224	1.985	0.960	0.968	0.041	0.057	0.049–0.065	596.544
Null model	7139.524	276	25.868	0.000	0.000	–	0.287	0.281–0.293	7187.524
1-Factor model	3444.940	252	13.670	0.490	0.535	0.156	0.205	0.199–0.211	3540.940
2-Factor model	2397.989	251	9.554	0.656	0.687	0.122	0.168	0.162–0.174	2495.989
4-Factor model	1517.552	246	6.169	0.792	0.815	0.060	0.131	0.125–0.137	1625.552
Measurement Model Sample 2	576.135	224	2.572	0.944	0.954	0.052	0.067	0.060–0.074	728.135
									
Structural Model Sample 2	626.129	241	2.598	0.942	0.950	0.058	0.067	0.061–0.074	744.129
Structural Model Sample 1	502.938	241	2.087	0.956	0.962	0.0464	0.060	0.053–0.067	620.938

*Recommended fit indices – relative chi square (χ^*2*^/*df*) ≤ 2, Tucker Lewis Index (TLI)≥0.90 or 0.95, Comparative Fit Index (CFI) ≥0.95, standardized root mean square residual (SRMR) < 0.08, root mean square error of approximation (RMSEA) ≤0.06 or 0.05, Akaike Information Criterion (AIC) lower values suggest better fit (Hu and Bentler, 1999; Kenny, 2015)*.

Given that measurement models often require re-specification (Anderson and Gerbing, 1988), and model parsimony is an important consideration for structural equation modeling (Bollen, 1989), modification indices were inspected to identify and retain the three highest-loading items for each construct. Jöreskog and Sörbom (1993) argued that a minimum of three items are required to define a construct.

The respecified CFA yielded improved and generally acceptable fit (see Table 1). Also, as shown in Table 1, the respecified measurement model provided superior fit relative to the null model, a one factor model, and an alternative four factor measurement model, with all 15 politics items loading on a single factor. Similarly, a theoretically defensible alternative two factor model, with all 15 politics items loading on a single factor and all meaning, engagement stress items loading on a single factor, did not provide acceptable fit. As shown in Table 2, all standardized loadings of the re-specified model were high (ranging from 0.679 to 0.982), and the five newly developed three-item politics scales demonstrated acceptable alpha reliabilities across both samples (ranging from α = 0.88 to α = 0.95). Reliability estimates for meaningful work, stress and engagement also exceeded the criterion standard for Cronbach’s alpha (ranging from α = 0.81 to α = 0.95). Furthermore, testing for common method variance (CMV) using procedures recommended by Podsakoff et al. (2012) showed that the decrease in standardized loadings ranged from 0.005 to 0.127 across the full set of 24 items included in the model. Furthermore, given that the average decrease across the 24 items was a very modest 0.06, and that all factor loadings remained statistically significant (*p* < 0.001) after the inclusion of the common method factor, the influence of method effects can, to a large extent, be discounted (Elangovan and Xie, 2000; Johnson et al., 2011; Podsakoff et al., 2012).

**Table 2:** Measurement model CFA standardized factor loadings and (alpha reliabilities).

Survey item		Sample 1	Sample 2
*Organizational Politics 1: Relationships* (Sample 1 α = 0.89; Sample 2 α = 0.90)			
	(1) People ingratiate themselves to other people to achieve the outcomes they desire.	0.892	0.892
	(2) People improperly use their relationships to bypass organizational processes.	0.876	0.882
	(3) People cultivate relationships in order to get personal benefits.	0.798	0.815
*Organizational Politics 2: Communication* (α = 0.92; α = 0.93)			
	(1) Gossip drives the way that people interpret what goes on in this organization.	0.926	0.904
	(2) Gossip is the primary way in which information is shared.	0.904	0.913
	(3) Rumors are central to people’s understanding of what is happening in this organization.	0.847	0.907
*Organizational Politics 3: Reputation (*α = 0.94; α = 0.95)			
	(1) Individuals stab each other in the back to make themselves look good.	0.916	0.925
	(2) People try to make themselves look good by making others look incompetent.	0.908	0.955
	(3) People undermine others’ credibility behind their backs.	0.931	0.926
*Organizational Politics 4: Decisions (*α = 0.90; α = 0.88)			
	(1) People use their position to influence decisions to benefit themselves	0.930	0.943
	(2) People abuse their authority by making decisions that benefit themselves.	0.945	0.937
	(3) People pretend to consult and invite input even though decisions have already been made.	0.739	0.679
*Organizational Politics 5: Resources* (α = 0.92; α = 0.89)			
	(1) People build up resources to increase their personal power, not to benefit the organization.	0.854	0.815
	(2) Too often, people unfairly obtain resources that could be better used elsewhere.	0.895	0.886
	(3) Resources are unfairly allocated based on individual influence rather than organizational priorities.	0.918	0.869
*Meaningful Work:* (α = 0.95; α = 0.92)			
	(1) The work I do in this job is very important to me.	0.857	0.854
	(2) My job activities are significant to me.	0.942	0.898
	(3) The work I do on this job is meaningful to me.	0.982	0.932
*Organizational Stress:* (α = 0.85; α = 0.81)			
	(5.1) If I had a different job, my health would probably improve.	0.790	0.751
	(5.2) I get irritated or annoyed over the way things are going here.	0.817	0.763
	(5.3) I seem to tire quickly.	0.804	0.794
*Engagement: (*α = 0.91; α = 0.88)			
	(5.1) When I get up in the morning I feel like going to work.	0.867	0.789
	(5.2) At my job I feel strong and vigorous.	0.881	0.874
	(5.3) I am enthusiastic about my job.	0.892	0.854

At the next stage of the analysis, the respecified CFA was run using Sample 2 data. Even though the measurement model again yielded acceptable fit (see Table 1), cross-validation procedures were used to more rigorously test the statistical equivalence or invariance of the 24-item measurement model across both samples. As a first step in the process (Bollen, 1989), the baseline test of the two-group model provided acceptable fit to the data (χ^2^ = 1,020.67, *df* = 448, CFI = 0.96, RMSEA = 0.044), thereby suggesting equivalence of form across the samples. Next, constraining the loadings to be equal across the samples resulted in a non-significant change in chi-square relative to the baseline model (Dχ^2^ = 24.986, *df* = 24, *p* > 0.05). Then, after additionally constraining the covariances to be equal, there was also a non-significant change in chi-square (Dχ^2^ = 24.058, *df* = 28, *p* > 0.05). Although, as a final step, after additionally constraining the error variances to be equal resulted in a significant change in chi-square (Dχ^2^ = 102.685, *df* = 24, *p* = 0.000), Byrne (2004) argued that constraining errors is unduly restrictive and an overly strict test of invariance. Overall, the invariance tests supported the generalizability of the model across the two samples.

Table 3 shows the means, standard deviations, interrater agreement (r_WG(J)_), and bivariate correlations among the first-order variables included in the 21-item respecified CFA for both samples. The *r*_WG(J)_ statistics (James et al., 1993), ranging from 0.44 to 0.62, indicate only low to moderate levels of agreement for the politics subscales in the Sample 2 data. The results therefore do not clearly support the ‘shared’ organizational level perceptions of organizational politics. The correlations in Table 3, however, show that most of the correlations were significant and in their predicted direction. The significant correlations between the politics scales and engagement and stress provided preliminary support for the proposed modeling. Contrary to expectations, however, a number of the correlations between the first order politics factors and meaningful work were not significant in Sample 2, and, although significant in Sample 1, were low.

**Table 3:** Means, standard deviations, interrater agreement (*r*_WG(J)_), correlations Sample 1 (below diagonal) and Sample 2 (above diagonal).

Measure	Mean sample 1	SD sample 1	Mean sample 2	SD sample 2	r_WG(J)_ sample 2	1	2	3	4	5	6	7	8
(1) Relationships	4.81	1.36	4.39	1.47	(0.62)	–	0.66	0.78	0.78	0.82	*-0.06*	0.45	-0.26
(2) Communication	3.96	1.63	3.75	1.56	(0.57)	0.66	–	0.64	0.64	0.71	-0.17	0.55	-0.39
(3) Reputation	3.83	1.64	3.70	1.69	(0.47)	0.68	0.66	–	0.84	0.73	-0.15	0.54	-0.31
(4) Decisions	4.25	1.52	3.84	1.59	(0.44)	0.74	0.62	0.78	–	0.79	*-0.09*	0.48	-0.26
(5) Resources	3.92	1.54	3.92	1.47	(0.61)	0.77	0.65	0.71	0.78	–	*-0.09*	0.51	-0.26
(6) Meaningful work	5.47	1.36	5.24	1.32		-0.17	-0.17	-0.20	-0.16	-0.18	–	-0.26	0.66
(7) Stress	3.50	1.67	3.81	1.58		0.50	0.52	0.57	0.49	0.53	-0.30	–	-0.65
(8) Engagement	4.93	1.49	4.57	1.38		-0.33	-0.36	-0.41	-0.34	-0.36	0.69	-0.68	–

*Values in italics are non-significant at *p* < 0.05*.

Figure 1 shows organizational politics modeled as a higher order construct. Despite the relatively strong correlations among the first order factors (ranging from 0.635 to 0.839), the validity of higher order models cannot be assumed and needs to be assessed (Credé and Harms, 2015). The ‘Target Coefficient 2’ (TC_2_; Marsh, 1987) was used to assess whether the higher order politics factors adequately explained the covariation among the first order factors. The TC_2_ (TC_2_ = 0.973) supported the higher order modeling. Furthermore, the first order factor loadings on the higher order factor (ranging from 0.737 to 0.907) all exceeded the recommended level of 0.50 (Leach et al., 2008).

**Figure 2:**
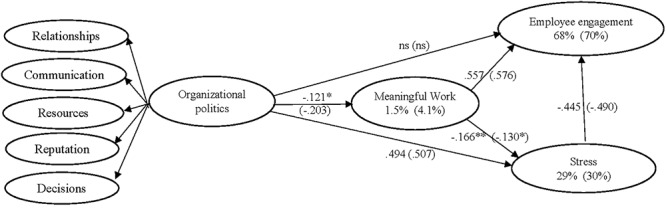
Proposed model standardized parameters; significant at *p* < 0.001 (unless ^∗^ < 0.05, ^∗∗^ < 0.01, or ns) and percent variance explained for Sample 2 (Sample 1 in parentheses).

Having established a defensible measurement model, the next step of the analyses involved testing the proposed structural relationships (see Figure 1). The fit indices showed the model fit the Sample 2 data reasonably well (see Table 1). With the exception of the relationship between politics and engagement, all of the proposed structural parameters were significant (see Figure 2). Although the relationship between the higher order politics factor and engagement was not significant, it is noteworthy that if the path from stress to engagement was deleted, the parameter from politics to engagement became significant (β = -0.260, *p* < 0.001). Furthermore, bootstrapping procedures established a significant indirect effect β = -0.319; *p* = 0.001) from politics to engagement through meaningfulness and stress. However, given that AMOS does not provide the significance of individual indirect effects, these tests were conducted in MPlus (Muthén and Muthén, 1998–2017). The analyses showed that organizational politics had a significant indirect effect on engagement through meaningful work (β = -0.11; *p* = 0.001; Confidence Interval 95%: -0.18 to -0.04) and through stress (β = -0.30; *p* = 0.001; CI95%: -0.40 to -0.21). Politics also had a significant indirect effect on stress through meaningful work (β = 0.04; *p* = 0.013; CI95%: 0.01 to 0.08); and meaningful work had a significant indirect effect on engagement through stress β = 0.094; *p* = 0.001; CI95%: 0.03 to 0.16). Overall, the model explained 4% of the variance in meaningful work, 68% of the variance in employee engagement, and 29% of the variance in individual stress. Additionally, invariance analysis demonstrated that the proposed structural model generalized across both samples (Dχ^2^ = 27.668, *df* = 26, *p* > 0.05). As such, the path coefficients were shown to be statistically equivalent across both samples.

## Discussion

As previously noted, the research aimed to test relationships between newly developed measures of organizational politics and two important aspects of the employee experience—employee stress and employee engagement. Furthermore, the research aimed to assess if work meaningfulness acts as a mediating mechanism to, in part, explain the relationships between organizational politics and the proposed outcomes.

Using data drawn from two independent samples and using quite stringent statistical tests, the results suggest a number of contributions to the literature. First, the results support previously reported direct effects of organizational politics on stress. Second, although oragnizational politics did not have a significant direct effect on engagement in either sample, politics were shown to have indirect effects on engagement through work meaningfulness and stress. These findings are important given the very considerable amount of research showing the important influence that both engagement and stress have on a range of individual and organizational performance and well-being outcomes. Stress, for example, has been linked to decreased employee health and well-being, increased turnover, higher absenteeism, and lower job performance (Randall and Perrewé, 1995; Summers et al., 1995), and employee engagement has been linked with organizational competitive advantage, job performance and employee well-being (Macey and Schneider, 2008; Crawford et al., 2010; Barrick et al., 2015). Consistent with previous research demonstrating that organizational politics, as a hindrance demand, can have an adverse impact on engagement (Crawford et al., 2010), the present study is therefore among the few to demonstrate that organizational politics has an influence, albeit indirect, on engagement. Organizational politics could therefore usefully be included as an organizational-level demand in future examinations of the Job Demands-Resources model (Bakker and Demerouti, 2007, 2014).

In addition to assessing the influence of politics on stress and engagement, the present research is one of the few to examine the influence of stress on engagement. Although demands are explicitly recognized as ‘stressors’ within JD-R research (e.g., Crawford et al., 2010), stress itself has not often been operationized within JD-R research. Instead, the majority of research looking at the health impairment pathway of the JD-R (Bakker and Demerouti, 2014) has focused on burnout or withdrawal behavior (e.g., Albrecht, 2015a). The finding that stress has a strong and direct effect on engagement suggests that stress too could usefully be included more explicitly in JD-R research models, and recognized as an important explanatory variable. The finding that stress mediated the influence of both organizational politics and meaningful work on engagement provides additional weight to its potentially important influence on engagement.

Further to the previous finding, and more generally, the research also makes a significant contribution to the literature by providing insight into how perceptions of organizational politics affect outcomes. Only a limited number of researchers have examined whether Kahn’s (1990) psychological conditions explain the relationship between politics and both engagement and stress. The results of the current research showed that work meaningfulness partially mediated the relationship between organizational politics and stress, and between politics and engagement. The current study also adds to the literature by being the first to examine the relationship between organizational politics and meaningful work. The results demonstrate that the negative use and abuse of relationships, communication channels, resources, reputation, and decision-making, all adversely impact employees’ perceptions that the work they do is meaningful and that through their work they make a meaningful contribution. It needs to be noted that although the influence of politics on meaning was significant, the association was not strong. Nevertheless, along with previous research showing that meaningful work is associated with psychological well-being (Zika and Chamberlain, 1992), positive mood (King et al., 2006), psychological benefits (Britt et al., 2001), and organizational commitment, job satisfaction, and job involvement (Milliman et al., 2003) this is an important finding as it identifies organizational politics as a potential ‘upstream’ antecedent of engagement.

The research introduced new measures of organizational politics. In support of the construct validity of the measures, the measures were shown to provide good fit to the data, to have acceptable reliabilities, and to be invariant across two independent samples. In further support of the measures, the strong and positive correlations between each of the five dimensions and stress correspond closely to previously reported meta-analytic associations between POPS and stress. Additionally, the measures have the advantage of being relatively brief. The research also contributes to the literature by showing the psychometric defensibility of a three-item measure of engagement. Schaufeli et al. (2017) argued “there is increasing pressure on researchers to develop valid, reliable, yet short measures without redundant items” (p. 2) to reduce the ‘burden’ placed on participants who are asked to complete organizational surveys.

Contrary to expectations, and the limited number of studies that have reported shared perceptions of organizational politics (e.g., Vashdi et al., 2013), the findings did not support the conceptualization of politics as a climate level construct in the Sample 2 data. The statistical tests of agreement did not reach generally accepted standards. By way of explanation, and consistent with the findings of Treadway et al. (2005a), the results may therefore suggest sub-climates within different organizational units or Divisions that explain the absence of strong shared perceptions across the sample. Alternatively, from a statistical perspective, Woehr et al. (2015) argued that agreement indices are likely to be lower at the organizational level relative to the group or team level of analysis. Irrespective of the explanation, Landells and Albrecht (2015) suggested that a diversity of perspectives about organizational politics within an organization is of as much interest as their sharedness. In practical terms, however, given the variability of organizational-level agreement about organizational politics across different studies, interventions to remediate organizational politics should be targeted at particular units or groups where it can be demonstrated that organizational politics is prevalent.

In terms of additional research opportunities, further research could usefully be directed toward identifying the individual and organizational variables that influence organizational politics. Given the influence that politics has on meaningful work, stress, engagement and other important outcomes, it will be useful to identify and incorporate influential upstream variables in research models. In a meta-analysis of the antecedents of organizational politics Atinc et al. (2010) identified the importance of organizational design characteristics such as centralization and procedural fairness. Atinc et al. (2010) also identified job and work environment characteristics such as autonomy, feedback, advancement opportunities, development opportunities, met expectations, trust, and leader-member exchange as important antecedents of politics. Atinc et al. (2010) also acknowledged the need for more research on the moderators of politics-outcome relationships (see for example Rosen and Hochwarter, 2014). However, given the number of years since Atinc et al.’s (2010) meta-analysis was published, and the relatively modest number of samples included in their analysis, additional research in more contemporary work contexts that includes the factors they identified could usefully be undertaken. More generally, given the scale and pace of change characteristic of the contemporary working context (van Dam, 2017), additional research could usefully be focused on identifying the influence of uncertainty, insecurity and change on the emergence and maintenance of organizational politics, work meaningfulness, stress, and engagement (e.g., DeGhetto et al., 2017). Similarly, the influence of more agile ways of organizing work on the emergence and nature of organizational politics could usefully be researched. Further research could also examine the role of psychological safety and psychological availability as mediators or moderators of the relationship between perceptions of organizational politics and outcomes such as engagement and stress.

A number of study limitations need to be acknowledged. Given the data were self-reports taken at one point in time the possible influence of common method bias needs to be considered. Although researchers have argued that the risks of common method are overstated (Conway and Lance, 2010), the use of quite rigorous CFA measurement techniques and cross-validation procedures conducted across the two samples helped establish the robustness of the findings. Furthermore, given the very modest average reduction in the standardized loadings after a common methods factor was included, and given that all the factor loadings remained statistically significant after the common methods factor was modeled, the issue of CMV appears not to be overly problematic. Nevertheless, cross-sectional studies do not permit interpretation of causality. Future longitudinal research could usefully be conducted to determine cause and effect relationships. This is particularly the case when testing mediation models (Maxwell et al., 2011; Kline, 2015). Caution also needs to be undertaken with regard to the generalizability of the findings. Both samples consisted of mostly Australian employees from either a public service agency or a range of public and private organizations. As past research has demonstrated that organizational politics perceptions are higher in public organizations (Vigoda-Gadot and Kapun, 2005), the findings need to be verified across additional organizational and cultural settings. Despite the limitations, however, the study has delivered a number of insights into the politics of organizations and presents clear opportunities for future research. Using the newly developed measures, this study confirmed that organizational politics has significant effects on stress and engagement. Furthermore, the explanatory power of work meaningfulness as a mediator of relationships with perceptions of organizational politics was also demonstrated. We look forward to future studies which validate this suite of measures in a range of cultural and contemporary organizational settings that further investigate the increasingly important construct of organizational politics.

## Ethics Statement

The research was approved by both authors’ university ethics committee. The approval was granted in accord with the Australian Government National Statement on Ethical Conduct in Human Research (2007). Before being able to proceed to the on-line survey, all participants clicked a response button confirming they understood the information provided in a participant sheet and confirming they consented to participate in the research. The participant information sheet made clear the anonymity and confidentiality of all responses. No inducements were provided.

## Author Contributions

All authors listed have made a substantial, direct and intellectual contribution to the work, and approved it for publication.

## Conflict of Interest Statement

The authors declare that the research was conducted in the absence of any commercial or financial relationships that could be construed as a potential conflict of interest.
